# Electromyographic Activity of the Pelvic Floor Muscles and Internal Oblique Muscles in Women during Running with Traditional and Minimalist Shoes: A Cross-Over Clinical Trial

**DOI:** 10.3390/s23146496

**Published:** 2023-07-18

**Authors:** María García-Arrabé, Pablo García-Fernandez, María José Díaz-Arribas, Jose Javier López-Marcos, Ángel González-de-la-Flor, Cecilia Estrada-Barranco, Jean-Sébastien Roy

**Affiliations:** 1 Faculty of Sport Sciences, Universidad Europea de Madrid, Villaviciosa de Odón, 28670 Madrid, Spain; maria.gararrabe@universidadeuropea.es (M.G.-A.); cecilia.estrada@universidadeuropea.es (C.E.-B.); 2 Department of Radiology, Rehabilitation and Physiotherapy, Faculty of Nursing, Physiotherapy and Podiatry, Complutense University of Madrid, 28040 Madrid, Spain; pablga25@ucm.es (P.G.-F.); mjdiazar@med.ucm.es (M.J.D.-A.); josejalo@ucm.es (J.J.L.-M.); 3 Department of Rehabilitation, Faculty of Medicine, Université Laval, Quebec City, QC 2325, Canada; jean-sebastien.roy@fmed.ulaval.ca

**Keywords:** minimalist shoes, pelvic floor, running, women’s sports, EMG

## Abstract

The study aimed to investigate the effects of footwear on the electromyographic (EMG) activity of pelvic floor muscles (PFMs) and internal oblique (IO) muscles during running at different speeds. The study also aimed to explore the correlation between EMG activity of PFMs and IO muscles and participants’ morphological characteristics. Ten nulliparous female runners were included in the study. The participants ran for 90 s at speeds of 9, 11, and 13 km/h wearing both traditional and minimalist shoes. EMG outcomes were presented as a percentage of maximum voluntary contraction (%MVC). Comparative analysis was conducted using the Wilcoxon rank test. Correlational analysis was performed using the Rho–Spearman correlation coefficient. The %MVC for the IO muscles was significantly lower when using minimalist shoes compared to traditional shoes (*p* = 0.04). No statistically significant differences were found for the PFMs (*p* > 0.05). The study also observed large correlations between age and %MVC of the PFMs and IO muscles (rho = −0.64; *p* = 0.04). Minimalist shoes decreased the activity of IO muscles in female runners. However, no significant differences in EMG activity of PFMs were found when comparing traditional and minimalist footwear. The long-term effects of minimalist footwear on EMG activity of PFMs and IO muscles, as well as their relationship to morphological characteristics, require further investigation.

## 1. Introduction

Stress urinary incontinence (SUI) is defined as the involuntary loss of urine during physical exercise or exertion, such as coughing or sneezing [1]. It is the most common form of UI in the female population and greatly affects athletes [2]. Multiple risk factors are associated with SUI: pregnancy, vaginal delivery, operations in the pelvic region, being overweight, and age [3]. High-intensity physical activity has also been shown to increase the risk of SUI in women [4]. However, studies are limited in terms of risk factors for a population of young nulliparous women. Increased abdomino-pelvic pressure has been proposed as a potential cause. High-impact physical activities, where both feet are no longer in contact with the ground at the same time, such as jumping or running, lead to an abrupt and repeated increase in abdominal pressure as well as an overload of the entire pelvic floor muscles (PFMs) [5].

The pelvic and abdominal cavities are linked biomechanically as they form a hydropneumatic cell with modifiable internal pressure [6]. Pressure in the abdominal cavity is determined by combined action of the PFMs, the abdominal musculature (e.g., internal oblique (IO)) muscles and the diaphragm [7]. Although SUI is frequently observed in physically active women, its aetiology and pathophysiology are still poorly understood. Several studies postulate that synergistic activation of abdominal muscles during PFM contraction is an essential mechanism for continence during exercise [8]. 

Running technique is influenced by footwear [9]. Running shoes can be classified as conventional or minimalist, with numerous studies investigating the effect of both on different aspects of running such as biomechanics, performance, or risk of injury [10,11,12,13,14]. Minimalist shoes are defined as shoes that provide minimal interference with the natural movement of the foot due to their high flexibility, low drop, low weight, minimal sole thickness, and the absence of technological devices [15]. To categorize footwear as minimalist, the Minimalist Index (MI) scale was created, establishing a minimum score of 70% for a shoe to be considered minimalist [16]. 

Minimalist footwear has been shown to have a positive impact on the strengthening of intrinsic foot musculature, improving force absorption and movement control. Inadequate foot muscle strength has been linked to a range of loading-related injuries [17]. Therefore, minimalist shoes may provide heightened stimulation to foot and leg muscles, thereby reducing joint load and potential injury risks [18]. The use of minimalist shoes has also been linked to changes in running technique (increased cadence, shorter strides, and forefoot stride) [19,20] and to improved running efficiency compared to the traditional shoe [13]. However, few studies have been conducted to explore the association between footwear and its impact on the activation patterns of the abdomino-pelvic complex, which includes the pelvic floor muscles (PFMs) and the abdominal muscles, including the internal oblique (IO) muscles [15]. PFMs play a crucial role in supporting pelvic organs and maintaining continence. Weakness or dysfunction in these muscles can lead to various PFM disorders, such as urinary incontinence or pelvic organ prolapse.

Despite the high prevalence of SUI in female athletes [21,22,23,24] and the fact that footwear-conditioned shock absorption is key to regulating and distributing running loads across joints and muscles of lower extremity [10,25,26], to date no studies have assessed the influence of footwear on PFMs and IO muscles during running. Surface electromyography (sEMG) provides information on how muscles operate to produce force; however, it is a measure of electrical activity emitted by a muscle and not a measure of force [27]. In fact, there is a large inter-subject variability in sEMG which could be explained by the fact that contraction characteristics can be affected by factors such as muscle mass, fat percentage, physical activity, height, etc. [28,29,30,31]. However, the specific relationship between surface electromyographic (sEMG) activity of the pelvic floor muscles (PFMs) and the internal oblique (IO) muscles and various morphological characteristics has not been thoroughly examined in previous studies. By examining sEMG activity differences between groups, we can gain insights into the muscular strength and function of the pelvic floor and the core. This information has practical implications in clinical settings, as it can help in diagnosing and managing PFM disorders and designing targeted rehabilitation programs. Additionally, in athletic and sports performance contexts, understanding the muscular differences between groups can provide valuable information for optimizing training programs, injury prevention strategies, and enhancing overall performance.

Therefore, the primary aim of the study is to analyze the sEMG activity of PFMs and IO muscles during running at different speeds (9, 11, and 13 km/h) and with different shoes: minimalist and traditional. A secondary aim is to explore the correlation between muscular sEMG activity and morphological characteristics of the participants (height, weight, and age). We hypothesized that PFM and IO sEMG is different during running with minimalist and traditional shoes and that morphological characteristics are associated with sEMG activity.

## 2. Materials and Methods

### 2.1. Study Design

Using a cross-sectional design, PFM and IO sEMG activity was collected during running at 9, 11, and 13 km/h with two types of shoes: conventional and minimalist. As in previous research, to capture the behavior of the musculature across different running conditions, we established three velocities that represented low, moderate, and high speeds [5]. This approach allowed us to observe muscle response across these three specific conditions.

The study was approved by the ethics committee of Hospital Clinical San Carlos (code 19/570-E_TFM) (Madrid, Spain). All subjects gave written informed consent.

### 2.2. Participants

Participants were divided into two groups: Group 1 comprised individuals who initially ran wearing traditional shoes and subsequently transitioned to minimalist shoes following a 10 min washout period. Conversely, Group 2 followed the reverse protocol. Random allocation assigned five participants to Group 1 and five to Group 2. This methodological approach aimed to mitigate potential confounding factors, such as fatigue, that could influence the outcome. Female recreational runners meeting the following inclusion criteria were recruited for the study: aged between 20 and 38 years [32], with a body mass index (BMI) ranging from 20 to 30 kg/m^2^, nulliparous, free from any pain or movement limitations during running, and capable of running on a treadmill at the predetermined target speeds. Exclusion criteria were pregnancy, any urogynaecological dysfunction, urogynaecological and lower limb surgeries performed in the last six months, and not being able to perform standardized voluntary contractions of the PFMs according to the Modified Oxford Scale. 

### 2.3. Sample Size Calculation

The sample size calculation was carried out using G*Power 3.1.9.2 software (G*Power©, University of Dusseldorf, Dusseldorf, Germany). Based on previous research conducted by Luginbuehl et al. [33] on the same population, a two-tailed hypothesis, with effect size: 0.80, α error probability = 0.05, and statistical power = 0.90, was employed for sample size calculation. A sample size of n = 10 was obtained.

### 2.4. Instrumentation

Running was performed on a Mercury model HP Cosmos treadmill (HP/Cosmos Sport & Medical, Nussdorf-Traunstein, Germany). During running, participants wore two types of shoes: minimalist and traditional shoes. The minimalist shoes, Vivobarefoot Primus Lite III (Vivobarefoot, London, UK), have a minimalist index of 84% (weight 181 g, drop 0 mm, high longitudinal and torsional flexibility, not equipped with any technological devices, and heel thickness of 10 mm). The traditional shoes, Sollomensi (Sollomensi, Guangzhou, China), have a minimalist index of 34% (weight 214 g, drop 20 mm, high resistance to longitudinal bending and to torsion, four technological devices (midsole, calcaneal reinforcement, raised medial insole, and sole widening), and heel thickness of 30 mm).

**Figure 1:**
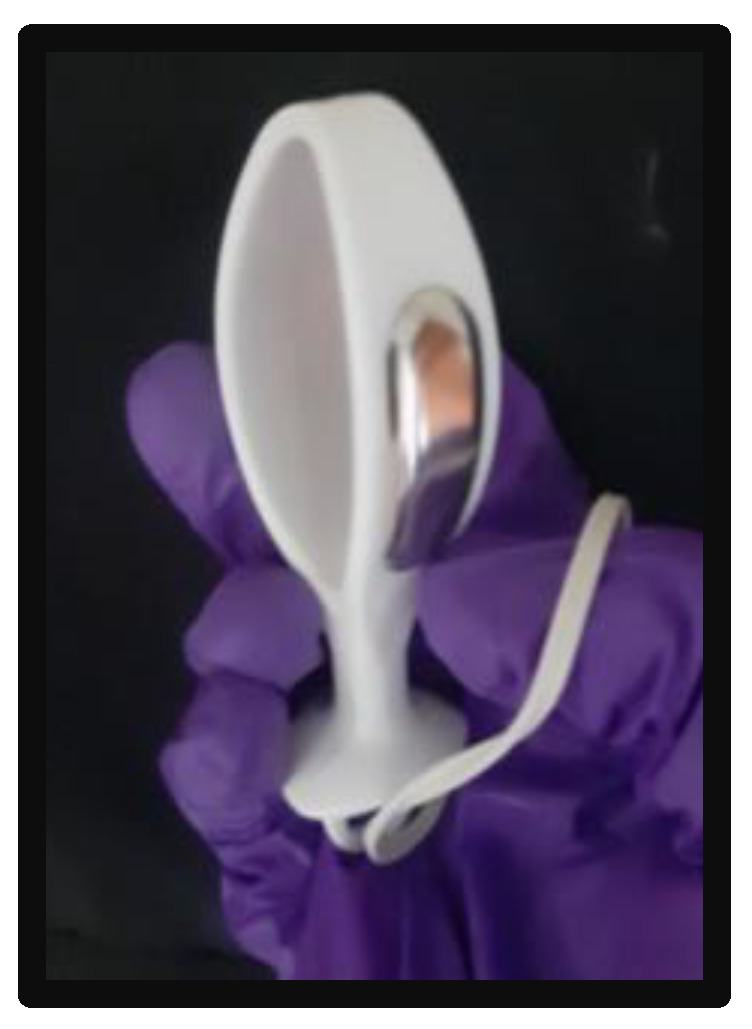
PeriformTM vaginal probe.

A PeriformTM vaginal probe (Neen HealthCare, Dereham, UK) connected to Neurotrac was used to measure EMG activity of the PFMs (Figure 1). The probe is 7.5 cm long in length, has a circumference of 10 cm, and has a monopolar configuration. It features two plates of longitudinal register (1.5 cm wide and 3.5 cm long) located in the body of the probe. Maximum voluntary contractions (MVCs) of the PFMs were measured in the supine position, with the participants having a pillow under their head. The lumbar spine was in neutral position with knees and hips slightly flexed, supported by a pillow under their knees. After electrode application, the participants performed one trial of different contractions (phasic, tonic, and endurance) to be familiarized with the testing procedures. Participants were then instructed to realize two maximum voluntary PFM contractions, each lasting 5 s, with a 1 min rest between contractions. 

**Figure 2:**
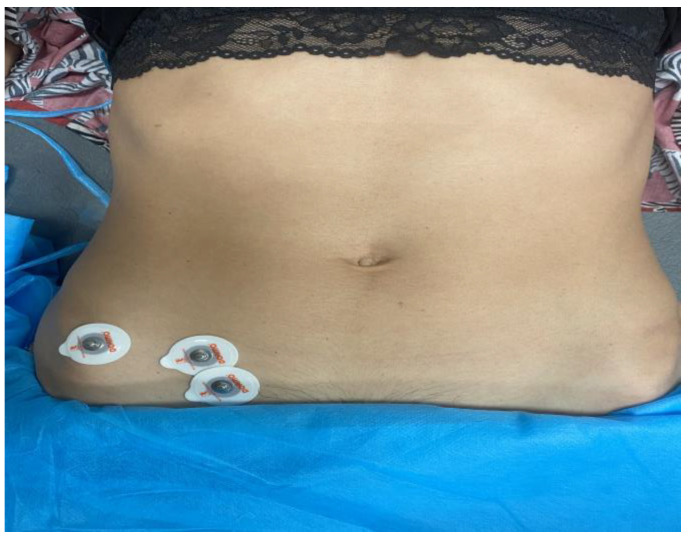
Electrode positions for the recording of electromyographic activity of the internal oblique muscle.

The IO muscle was located by palpation during submaximal isometric contraction, and sEMG electrodes (Natus Medical Incorporated, Middleton, CA, USA) were placed according to the reference positions: in the triangle formed by the inguinal ligament, the anterior superior iliac spine, and the line umbilical stocking; additionally, an electrode was placed as a reference electrode on the anterior superior iliac spine [33,34] (Figure 2). The participants were placed in a supine position and were instructed to rotate their trunk during the testing measurement. Participants were instructed to realize two MVCs of the IO muscles, each lasting 5s, with a 1 min rest between contractions. The EMG system used was a Step 32^®^ (Trimedica, Madrid, España). A ground electrode and amplifier were used to register EMG activity. The EMG signal was amplified from a minimum of 1000 to a maximum of 50,000 times with a total noise referred to the lower input 22 nV/Hz rms, mainly due to opposition noise of the skin/electrode. These features allow the recording of electromyographic signals in critical conditions, such as in patients with reduced muscular mass and very thick adipose panniculus. Band-pass filtering was applied to the sEMG signals during data processing. The purpose of this filtering was to remove any potential noise or interference outside the relevant frequency range of the muscle activity. Although the upper cutoff frequency mentioned in Table 1 may seem high, it was chosen based on the specific characteristics of the sEMG signals and the frequency content associated with the studied muscle activity.

**Figure 3:**
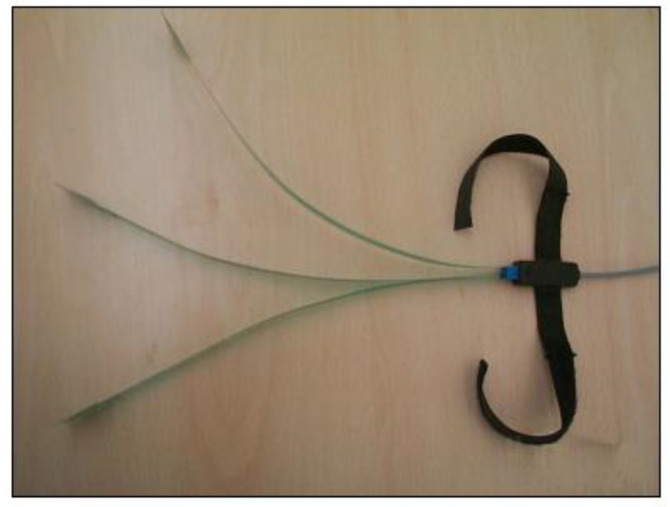
Basographic sensors.

The basographic sensors (Figure 3) employed in our study were composed of a shape-switching rectangular component, approximately 11 mm on each side. These sensors were placed at the end of a flexible tape made of insulated plastic material. The purpose of these sensors was to record the gait cycle in relation to the electromyographic (EMG) signal. At the opposite end of the sensor, a connector was applied. This connector was necessary for connection with the preamplifier/decoder, which allowed us to obtain and process the signals generated by the basographic sensors.

### 2.5. Procedures

Participants in this study were randomly distributed into two groups according to the sequence of the evaluation. Group 1: a 5 min run on the treadmill at their preferred speed to warm up, followed by a 30 s [33,35,36] run at each speed (9, 11, and 13 km/h) with traditional shoes. After a 10 min washout period during which participants sat down, they performed the same protocol with minimalist shoes. Group 2: a 5 min run on the treadmill at their preferred speed to warm up, followed by a 30 s run at each speed (9, 11, and 13 km/h) with minimalist shoes. After a 10 min washout period during which participants sat down, they performed the same protocol with traditional shoes. Random assignment of the order of the sequence of shoes was made based on the table of random permutations from Moses and Oakford [37]. Five participants were included in each group.

### 2.6. Data Reduction

EMG signals of PFMs were sampled using the software package Neurotrac^®^ (NeuroTrac ETS; Verity Medical, Romsey, UK), and the EMG system used for the IO muscles was a Step 32^®^ (Trimedica, Madrid, España). To ensure accurate synchronization between the Neurotrac device and Step 32, we employed a timer-based approach. Both devices were connected to a centralized timing system that served as the master timer. This master timer was programmed to send a synchronization signal simultaneously to both the Neurotrac and Step32 devices. By using this synchronized timing system, we ensured that data collection from both devices occurred simultaneously. This approach allowed us to accurately correlate the measurements obtained from the Neurotrac device, which captured muscle activity, and the Step 32 device, which recorded gait parameters.

The sampling frequency for EMG signals was set at 1000 Hz. The selected sampling frequency allowed us to capture the necessary information from the EMG signals while balancing the trade-off between data resolution and storage capacity. EMG signals were registered for 90 s in each participant, and every 30 s was at a different speed. The first 10 s of each velocity (9, 11, and 13 km/h) were discarded because the treadmill needed between 3 and 4 s to reach the target speed set by the protocol. Therefore, the EMG data of each participant were averaged over 20 s at each speed. The following variables were selected from the EMG analysis: work average (µV), the overall average of microvolts achieved during all the work periods of the session (60 s), and mean percentage of MVC (%MVC). 

### 2.7. Statistical Analysis

Statistical analysis was performed using SPSS 25.0v, with the α error adjusted at 0.05. To examine data distribution, the Shapiro–Wilk test was conducted. Mean ± standard deviation (SD) was used to represent parametric data (Shapiro–Wilk test with a *p*-value ≥ 0.05), along with the range (minimum–maximum). For non-parametric data, the median ± interquartile range (IR) was used, and completed with the range (minimum–maximum). Furthermore, EMG differences between shoes were evaluated using the paired *t*-test for parametric data and the Wilcoxon rank sum test for non-parametric data. The Rho–Spearman correlation coefficients were utilized to explore the correlation between the EMG variables and the participants’ characteristics. The magnitudes of correlation between variables were interpreted according to the following criteria: trivial (r ≤ 0.1), small (r = 0.1–0.3), moderate (r = 0.3–0.5), large (r = 0.5–0.7), very large (r = 0.7–0.9), and almost perfect (r ≥ 0.9) [38].

## 3. Results

A total of 10 participants were included in the present study. The characteristics of the total sample are presented in Table 2, which provides detailed information on anthropometric variables, such as age, weight, height, and BMI.

Data of the sEMG variables recorded for the IO muscles and PFMs are presented in Table 3. There was a significant statistical difference with large effect size between traditional and minimalist shoes in the %MVC of the IO muscles (*p* = 0.04; effect size = 0.77), where the minimalist shoes led to lower values.

Table 4 shows the correlation data between the EMG variables and age, height, and weight. A negative significant statistical correlation was observed between age and the average variables of IO sEMG activity with traditional shoes (*p* < 0.05; rho = −0.64). 

## 4. Discussion

The aim of this study is to describe and compare the short-term effects of electromyographic activity in PFMs and IO muscles during running in nulliparous women wearing minimalist versus conventional footwear. No differences were found in the EMG activity of PFMs when comparing the two types of shoes. However, activation of the IO muscles was lower when running with minimalist shoes. 

PFM EMG activity was not different when running with minimalist or traditional running shoes at different speeds in healthy nulliparous women. This lack of difference leads us to believe that an adaptation and training time to minimalist footwear may be necessary for plausible clinical modifications to occur [39]. Previous studies have reported that the first attempts at barefoot running do not guarantee a complete transition to a forefoot/midfoot support pattern [40]; this fact could explain the lack of significant differences in the EMG parameters recorded in PFM activation during the use of the two types of footwear. 

However, statistically significant differences were found for the IO muscle, in mean %MVC, which is lower when running with minimalist shoes. This finding is consistent with the idea that the use of minimalist footwear increases running efficiency [41]. By decreasing the energy expenditure derived from voluntary muscle contraction required during running, the use of minimalist shoes may have led to a modification of the recruitment of slow- and fast-twitch fibers [34]. It may provide an effective mechanism to reduce muscle fatigue and may be a preventive factor for PFM-overload dysfunction such as overtraining or fatigue-related SUI [29]. In addition, decreasing the amount of muscle work with minimalist shoes seems to favor synergy and reflex contraction with the PFMs [5], and could be a preventive factor for dysfunction in the abdomino-pelvic cavity that is so frequent in female runners. 

Age was largely correlated with average EMG activity of the IO muscles (rho = −0.64) when running with traditional shoes: the older the age, the lower the average recruitment. This coincides with previous research showing an increase in the prevalence of UI and dysfunction of muscular activity in older adults [42]. However, correlation between age and height with minimalist footwear was not significant.

Based on the results obtained in this study, running with minimalist shoes could decrease IO activity in women and may help to reduce symptoms in pathologies such as SUI or groin pain. Therefore, we consider that it would be of great importance to continue with this line of research, in order to deepen our knowledge of the influence of the use of different types of sports footwear on the EMG activity of PFMs and IO muscles, given that changes in the recruitment of muscle fibers associated with footwear during running may provide information for prevention and treatment in women. Future studies in which runners make a progressive transition to minimalist shoes would be interesting in order to study the changes that occur in the medium and long term in the musculature of the pelvic region. It would also be advisable to study muscle behavior in women with PFM dysfunction or in women who have had a vaginal delivery or caesarean section.

The present study has several limitations that should be acknowledged. First, the nature of the experiment precludes masking of therapists and patients. This limitation is inherent to the type of experiment being performed. Second is the absence of biomechanical analysis using, for example, pressure platforms, running cadence, or strike length. And third, sEMG provides valuable insights into muscle activity, but it has inherent limitations in spatial resolution. The electrodes used to capture muscle activity are typically placed on the surface of the skin, which can lead to signal contamination from neighboring muscles. This limitation may hinder the ability to accurately isolate and analyze the activity of specific individual muscles or small muscle groups, potentially reducing the precision of the findings. sEMG primarily captures the activity of muscles close to the skin surface, neglecting deeper muscles. This limitation can result in an incomplete understanding of overall muscle function, particularly in studies focusing on deep or underlying muscles. Additionally, the placement of surface electrodes may not accurately capture muscle activation patterns in deeper muscles, potentially limiting the generalizability of the findings to the entire muscle group or complex movement patterns. In our study, we chose to segment the sEMG signal into 20 s intervals for the purpose of characterizing the EMG activity of each muscle. We recognize that this segment duration may seem long compared to typical sampling frequencies of sEMG signals, which usually require sampling frequencies higher than 200 Hz.

However, we should point out that our intentions were to obtain a more complete and representative view of EMG activity over a longer period of time. By selecting a 20 s segment, we sought to capture both short-term variations and long-term trends in the sEMG signal. This approach allowed us to obtain a more global picture of muscle activity and its characteristics during the specific activity we were studying. In addition, to address the potential concern of bias due to segment duration, we implemented an approach of averaging the data over that time period. After extracting the two parameters of interest, we averaged these parameters over each 20 s segment. In doing so, we sought to reduce any random influence or variability that may be present within the segment.

It is important to acknowledge these limitations when interpreting the results of electromyographical studies and to consider alternative methods or approaches to overcome these challenges for a more comprehensive understanding of muscle activity. However, these limitations do not prevent the study from being of the highest methodological quality, creating a cross-over design to avoid a lack of homogeneity in the two groups. In this study, the randomization of the sequence of intervention on the patients and the analysis of the results was blinded. 

## 5. Conclusions

Our findings suggest that nulliparous female runners wearing minimalist shoes exhibit lower electromyographic activity of the IO muscle compared to those wearing traditional shoes in 90 s of running. No differences were found in the EMG activity of PFMs comparing the two types of shoe. Further research is needed for pelvic floor dysfunction in the relationship between PFMs and IO muscles in nulliparous female runners.

## Figures and Tables

**Table 1:** Characteristics of EMG sensors.

*Characteristics*	*Variable Geometry EMG Sensor* 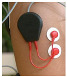	*Fixed Geometry EMG Sensor* 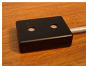
*Weight*	5 g	5 g
*Measurements*	8 × 37 × 21 mm	7 × 27 × 19 mm
*Distance between electrodes*	Variable	12 mm
*Electrode composition*	Single use	Ag or Ag-AgCl
*Differential amplification*	990 *v*/*v* ± 0.2%	990 *v*/*v* ± 0.2%
*Common mode rejection coefficient*	>113 dB	>113 dB
*Input impedance*	>1.1 GΩ	>1.1 GΩ
*Output impedance*	<100 Ω	<100 Ω
*Lower cutoff frequency*	20 Hz	20 Hz
*Upper cutoff frequency*	20.3 kHz	20.3 kHz
*Equivalent input noise*	<22 nV/Hz	<22 nV/Hz
*Supply voltage*	Of ± 2 V ± 6 V	Of ± 2 V ± 6 V
*Absorbed current*	±865 µA	±865 µA

EMG: electromyographic; mm: millimeters; V: volts; dB: decibels; GΩ: gigaohms; Ω: ohms; Hz: hertz; kHz: kilohertz; µA: microamperes.

**Table 2:** Participants’ anthropometric characteristics.

	Mean ± SD	Range (min–max)
Age	27.1 ± 5.3	20–36
Weight	64.9 ± 1.4	58–80
Height	168 ± 5.1	165–180
BMI	22.8 ± 2.5	20.0–28.3

Abbreviations: BMI: body mass index; SD: standard deviation.

**Table 3:** Effects of shoes on electromyographic variables in the IO muscles and PFMs.

	EMG Variables	Minimalist Shoes n = 10	Traditional Shoes n = 10	Mean Difference (95%CI)	*p*-Value (ES)
*IO*	work average (µV)	14.6 ± 6.9	15.6 ± 8.2	−1.0 (−6.0;8.0)	0.21 (0.10)
	% of MVC	16.7 ± 10.8	21.2 ± 10.1	−4.4 (0.03;8.9)	0.04 (0.77)
*PFM*	work average (µV)	31.7 ± 10.4	31.9 ± 9.8	−0.2 (−3.9;4.4)	0.33 (0.04)
	% of MVC	36.3 ± 4.8	34.7 ± 4.5	1.6 (−6.0;8.0)	0.23 (0.50)

Abbreviations: CI, confidence interval; ES, effect size; IO, internal oblique muscle; PFM, pelvic floor muscle; MVC: maximum voluntary contraction. Significance was set at *p* < 0.05. Data are expressed as mean ± standard deviation.

**Table 4:** Correlation analysis between the electromyographic recording of the IO muscles and PFMs and the variables of height, weight, and age.

Muscles	Variables		Height	*p*	Weight	*p*	Age	*p*
**IO**	Traditional Shoes	work average (µV)	0.08	0.81	−0.8	0.8	−0.64 *	0.046
		% of MVC	0.62	0.07	−0.2	0.52	−0.25	0.84
	Minimalist Shoes	work average (µV)	0.61	0.06	0.12	0.73	−0.04	0.24
		% of MVC	0.49	0.17	−0.05	0.88	−0.41	0.27
**PFMs**	Traditional Shoes	work average (µV)	−0.23	0.51	0.18	0.61	0.28	0.43
		% of MVC	−0.34	0.36	−0.48	0.18	0.01	0.9
	Minimalist Shoes	work average (µV)	−0.19	0.59	−0.03	0.92	0.53	0.11
		% of MVC	−0.38	0.3	0.07	0.84	0.08	0.98

IO: internal oblique; MVC: maximum voluntary contraction; PFMs: pelvic floor muscles; Spearman correlation coefficient was performed. * *p* < 0.05.

## Data Availability

The data presented in this study are available on request from the corresponding author. The data are not publicly available due to privacy and ethical restrictions.
